# Mr. Watkinson, on Inversio Uteri

**Published:** 1802-05

**Authors:** Henry Watkinson

**Affiliations:** Ashton, near Warrington


### To Dr. BATTY.

SIR,

IF you fhould deem the following worthy a place in your valuable Journal, you will much oblige me by inferting it.

*Ashton, near Warrington,* I am, &c.

*March 3, 1802.* HENRY WATKINSON.

---

THE Inverfio Uteri is a complaint that is noticed by moft writers on Midwifery, as fometimes occurring after or along with the extraction of the placenta, and as ufually very foon terminating fatally, if not immediately reduced, and even then as being attended with the greateſt danger. But few authors, and I believe none of our late ones, on either Midwifery or Surgery, have mentioned the diſeaſe as occurring at a different period, originating from a different cauſe, and exiſting for a long period of time in an inverted and prolapſed ſtate without proving fatal. Some of the older writers have, I believe, mentioned it, and have even ventured to amputate the part, but the fatal termination of which has deterred others. A few years ſince a caſe occurred, which is related in Duncan's Annals of Medicine for 1799, where a tumour was attached to the fundus uteri, which, by degrees, inverted that organ, ſome days after labor, and which it was found impoſſible to reduce; the uterus, therefore, remained in that ſtate a fortnight, when a conſiderable diſcharge and hectic ſtate threatened the life of the patient; and it was found neceſſary to amputate it, which was performed, and the patient perfectly recovered.

In the courſe of my practice I have met with two caſes of Inverſio Uteri, both of which occurred at a period very diſtant from labor, and had continued ſo for a number of years. Of the hiſtory of the firſt I know little; it occurred to me while I was a reſident of Ormſkirk, and I have not now an opportunity of making further inquiry about it. Of it, I only know, that the womb was completely inverted, about the ſize of a fiſt, hung pendulous without the os externum, but might be readily returned within it, in its inverted ſtate, but ſoon paſſed through again. The woman was about ſixty years of age, had had the complaint ſeveral years, was able to walk about, and even travel miles into the country; but there was a conſiderable thin diſcharge from the ſurface, which weakened and diſtreſſed her a good deal.

The ſubject of the ſecond caſe was the wife of Robert Scotſon of Lowton, in this neighbourhood. This woman had had the complaint about four or five years, and was about fifty-four years of age. It took place during a long protracted and very ſevere mænorrhagia about her fiftieth year.

On her firſt application to me, ſhe made me underſtand it to be a prolapſus, and I promiſed to relieve her by a peſſary; but on her ſending for me for that purpoſe ſome time afterwards, I found her laboring under a ſuppreſſion of urine, which had occaſionally occurred to her; and on examining the uterus, I found it completely inverted and about the ſize of my hand. It might readily be puſhed within the os externum, but on walking, ſoon came down again; and although it might be kept up, by means of a peſſary, which, for a time, made her much more comfortable and capable of walking, yet, the ſize of the tumor rendered rendered that inftrument of little ufe, as on the leaft exertion or exercife, its preffure (in that fituation) upon the urethra and bladder very foon brought on a return of the fuppreffion of urine. Under thefe circumftances, therefore, little could be done, except relieving occafional fymptoms, and I faw no more of her for fome months. At the end of this period I faw her again, and found that in the interim, a frefh train of fymptoms had taken place; from fome caufe, a violent inflammation had attacked the uterus, it had increafed to double the fize, and had become gangrenous in feveral parts. I now found it near the fize of a new-born infant's head, gangrenous and floughing in feveral parts, and the greateft part of its furface difcharging a copious fœtid fluid. It hung nearly half way down her thighs, and had a pretty long neck, about the thicknefs of my wrift, which was formed by the inverted vagina, and which, in one place, near its attachment to the uterus, had mortified through, and from which a very profufe hæmorrhage had taken place a few days before. My patient was now in a very low, weak, emaciated ftate, with hectic fymptoms; her pulfe was weak and frequent, her appetite gone, and fhe was almoft incapable of taking wine. She conftantly lay upon her back, and was not able, from the pendulous ftate of the tumor and her weak ftate, to be got up, even to anfwer the calls of Nature, and upon the whole fhe appeared to be declining very faft. I explained to her and her friends her dangerous fituation, and mentioned to them that amputation of the womb, although very dangerous and uncertain, was the only probable means of faving her life. Of this, they determined to confider a few days, when, during that time, a humane clergyman in the neighbourhood brought Dr. Mofs of Warrington, to fee her, who, concurring in what I had faid, propofed to meet me in a few days, that he might perform the operation. At the appointed day we accordingly met, and after lying her upon a table, on her back, we tied a ligature very firmly about half way of the neck, and placed another loofely a little higher up, to tighten if neceffary, and then with a fcalpel I divided its neck clofe to the uterus. We now proceeded to take up the divided veffels, but on a fudden the divided vagina, which had formed the neck of the tumour retracted, threw off both the ligatures, and returned itfelf into its original fituation, within the os externum. An hæmorrhage now took place, which Dr. Mofs endeavoured to reftrain by preffure within the vagina; in doing which, an abfcefs burft, and difcharged a quantity of very fœtid matter. The hæmorrhage by degrees abated, and although (I think) fhe did not lofe more than a pint of blood, yet, as it was arterial, and the patient too weak to bear fuch a difcharge, fhe became very faint; a cold cold clammy ſweat broke out, her pulſe was weak and tremulous, ſickneſs took place, and ſhe expired in about two hours.

Although the above caſe turned out ſo unfortunately, yet as a rare inſtance of the diſeaſe, I think it may be worthy of record; and although the practice offers nothing new, yet I think it may prove a caution to other practitioners, under like circumſtances, to take more pains to ſecure the neck of the tumour, formed by the inverted vagina, from retracting, before the veſſels are properly ſecured. Before the operation we had propoſed to paſs a firm ligature, by a needle, in a crucial manner, through the middle of the upper part of the neck, to ſecure the divided end the more certainly; but gave it up, as we conſidered the ligature round the neck to be ſufficient. If this plan had, however, been made uſe of, I am inclined to think that the hæmorrhage might have been prevented, and *perhaps* the life of the patient ſaved.

Of the cauſe and origin of the inverſion in this laſt caſe, I think there can be little doubt; it ſeems to have been occaſioned by the relaxed ſtate of the os uteri, and perhaps of the uterus itſelf, occaſioned by the long protracted hæmorrhage, and to be excited by the preſence of coagula (which muſt neceſſarily have formed during that period,) and along with ſome of which it might probably have become inverted.

P. S. Since drawing up the above ſtatement, I ſent a copy to Dr. Moſs for his peruſal and approbation; he has returned it, and ſays, " You have my full conſent to publiſh it, with my name as therein expreſſed;" and then adds, " I recollect that an artery which I had once taken hold of with the tenaculum, ſhewed no diſpoſition to be drawn out for tying; from which I ſuſpect, *at least*, there would be ſome difficulty in paſſing ligatures round the arteries of this part. How far might it be proper to apply the actual cautery in ſuch a caſe? I do not think the parts under theſe circumſtances are very ſenſible, the patient did not complain in the operation.

